# HVEM Promotes the Osteogenesis of allo-MSCs by Inhibiting the Secretion of IL-17 and IFN-γ in Vγ4T Cells

**DOI:** 10.3389/fimmu.2021.689269

**Published:** 2021-06-23

**Authors:** Lei He, Jun Xiao, Lei Song, Rui Zhou, Zhigang Rong, Weifeng He, Fei Dai

**Affiliations:** ^1^ Department of Orthopaedics, First Affiliated Hospital, Army Medical University, Chongqing, China; ^2^ Special Service Recuperation Center of Rocket Army, Guangzhou, China; ^3^ State Key Laboratory of Trauma, Institute of Burn Research, Southwest Hospital, Army Medical University, Chongqing, China

**Keywords:** HVEM-BTLA, Vγ4T cells, MSc, Tissue engineered bone, IL-17, immunomodulatory

## Abstract

Bone defects are a common orthopaedic concern, and an increasing number of tissue-engineered bones (TEBs) are used to repair bone defects. Allogeneic mesenchymal stem cells (allo-MSCs) are used as seed cells in many approaches to develop TEB constructs, but the immune response caused by allogeneic transplantation may lead to transplant failure. V gamma 4 T (Vγ4T) cells play an important role in mediating the immune response in the early stage after transplantation; therefore, we wanted to verify whether suppressing Vγ4T cells by herpesvirus entry mediator (HVEM)/B and T lymphocyte attenuator (BTLA) signalling can promote MSCs osteogenesis in the transplanted area. *In vitro* experiments showed that the osteogenic differentiation of MSCs and Vγ4T cells was weakened after co-culture, and an increase in interleukin-17 (IL-17) and interferon-γ (IFN-γ) levels was detected in the culture supernatant. HVEM-transfected MSCs (MSCs-HVEM) still exhibited osteogenic differentiation activity after co-culture with Vγ4T cells, and the levels of IL-17 and IFN-γ in the co-culture supernatant were significantly reduced. *In vivo* experiments revealed that inflammation in the transplanted area was reduced and osteogenic repair was enhanced after Vγ4T cells were removed. MSCs-HVEM can also consistently contribute to reduced inflammation in the transplanted area and enhanced bone repair in wild-type (WT) mice. Therefore, our experiments verified that HVEM can promote the osteogenesis of allo-MSCs by inhibiting IL-17 and IFN-γ secretion from Vγ4T cells.

## Introduction

Bone defects are common diseases treated in orthopaedic clinics, and most bone defects are caused by bone tuberculosis, osteoarthritis, bone tumour resection and severe fracture (1, 2). Autogenous bone grafts are the gold standard for the treatment of bone defects, but the lack of an autogenous bone graft source and risks of donor site infection and bleeding are major obstacles. Therefore, allogeneic bone transplantation has become the other choice of treatment, but it has the disadvantages of high cost and strong rejection (2). The development of tissue-engineered bone (TEB) derived from mesenchymal stem cells (MSCs) as seed cells have begun to emerge in the medical and scientific communities. Through tissue engineering, autologous bone marrow-derived MSCs (BMMSCs) were implanted into the scaffold material of TEB. This structure was found to actively assist in bone regeneration (2–5). The proliferation and differentiation ability of BMMSCs *in vitro* decreased with increasing donor age. In addition, it takes too long to construct autologous TEB grafts *in vitro*, and it is difficult for autologous MSCs to meet the needs of individual and large-scale clinical applications. Therefore, allo-MSCs have been used as seed cells by many researches aiming to develop ideal TEB constructs (6).

The success of allogeneic TEB depends on the strength of the immunogenicity induced by the transplanted cells. Although MSCs have low immunogenicity, they can still induce natural immune rejection when transplanted in animals (7–9). The inflammatory response induced by immune rejection will seriously affect the osteogenic differentiation of implanted MSCs and cause transplantation failure. In allogeneic transplantation, γδT cells modulate the size and productivity of pre-immune peripheral B cell populations and mediate early graft versus host disease (GVHD) (10, 11). V gamma 4 T cells (Vγ4T cells, a subtype of γδT cells) are the main producers of IL-17 during early GVHD; this production induces an early inflammatory response and plays a key role in the early innate immune response (4-96 hours) (11–15). IL-17 can inhibit the osteogenic differentiation of MSCs (16). Thus, we hypothesize that inhibiting the function of Vγ4T cells to reduce IL-17 secretion promotes the osteogenesis of TEB arising from MSCs.

B and T lymphocyte attenuator (BTLA) is an inhibitory receptor of the Ig superfamily that plays an important role in negative immune regulation (17). BTLA plays an active role in alleviating GVHD (18, 19). The BTLA protein structure is similar to that of programmed death 1 (PD-1) and cytotoxic T lymphocyte associated antigen 4 (CTLA-4) and comprises an extracellular domain, a cytoplasmic domain and a transmembrane domain (20). The unique ligand of BTLA is herpesvirus entry mediator (HVEM), which belongs to the tumour necrosis factor (TNF) receptor superfamily and is also widely expressed in B cells, T cells, macrophages, DCs, and endothelial cells. The discovery of HVEM and its receptor BTLA provided the third immunosuppressive pathway similar to PD-1 and CTLA-4. Studies have shown that BTLA signalling inhibits donor versus host T cell responses and improves GVHD *via* successful implantation of donor haematopoietic cells. Activation of the HVEM-BTLA signalling pathway can overcome lymphopenia and T cell proliferation after haematopoietic stem cell transplantation, thus preventing GVHD and host versus graft reaction (HVGR) without global immunosuppression and reducing the acute inflammatory response (18, 19). As the osteogenic differentiation ability of allo-MSCs was inhibited by IL-17 and IFN-γ (16, 21), the early source of IL-17 in the transplant area was identified as Vγ4T cells (12); thus, we investigated whether HVEM-BTLA signalling could inhibit the function of Vγ4T cells to promote allo-MSC-based TEB repair of bone fractures. First, we implanted TEB constructed with MSCs into wild-type (WT) mice and a Vγ4T cell-free femoral defect mouse model (Vγ4-D) to observe the osteogenic ability of the cells *in vivo*. Then, the osteogenic differentiation potential of MSCs or HVEM-expressing MSCs was measured when these cells were co-cultured with Vγ4T cells *in vitro*.

## Materials and Methods

### The Extraction, Proliferation and Identification of MSCs

The animal experiments were approved by the Animal Ethics Committee of the Third Military Medical University. Four-week-old male C57BL/6 mice (Experimental Animal Centre of Third Military Medical University, Chongqing, China) were killed, and their femurs and tibias were harvested under sterile conditions and rinsed with phosphate-buffered saline (PBS) before removal of all soft tissues still attached. The ends of the bones were cut off, and the bone marrow was washed out with C57BL/6 mouse MSC complete medium (Cyagen Biosciences, China). The resulting cell suspension was transferred to a 15 ml centrifuge tube (Nest, China) and centrifuged for 10 minutes at 200 ×g, after which the supernatant was discarded. The cell suspension was resuspended in MSC medium at a density of 1 × 10^6^ cells/ml, seeded into six-well plates and cultured at 37°C and 5% CO_2_. The medium was first changed the next day before shifting to a schedule of every 3 days until the cells reached 90% confluence. The cells were washed with sterile PBS three times, digested with 0.25% EDTA trypsin (HyClone, USA) at 37°C for 3 minutes, treated with medium to terminate the digestion, gently pipetted several times, and centrifuged for 10 minutes at 200 ×g; the supernatant was then discarded. The cells were resuspended in medium at a density of 1 × 10^5^ cells/ml and seeded in a six-well plate (NEST, China). MSCs were identified after the 3^rd^ passage (P3). P3 cells were digested, centrifuged and resuspended in PBS. MSCs were identified by flow cytometry with antibodies against CD11b, CD29, CD31, CD44, CD45 and CD90 (eBioscience, BioLegend, Abcam). P3 MSCs were infected with adenovirus carrying HVEM-EGFP and EGFP (kindly donated by Professor He Weifeng, Burn Research Institute of Southwest Hospital). The transfection efficiency was observed by fluorescence microscopy.

### Isolation, Culture and Identification of Vγ4T Cells

Eight-week-old female BALB/c mice were anaesthetized and sacrificed. After the mice were immersed in 75% alcohol for 3 minutes, the spleen was removed and ground into pulp in sterile PBS by glass sliding before the tissue was centrifuged for 10 minutes at 200 ×g; the supernatant was discarded. Two millilitres of sterile red cell lysis buffer was added for 1 minutes to lyse residual red blood cells before 10 ml of 1640 medium was added to terminate the reaction. The samples were centrifuged at 200 ×g for 10 minutes, and the supernatant was discarded. Vγ4T cell culture medium comprised 1640 medium (Hyclone, USA), CD28 antibody (BioXcell, USA), rIL-2 (R&D Systems, USA), β-mercaptoethanol (Sigma, USA), and 10% foetal bovine serum (Gibco, USA), penicillin-streptomycin solution (Beyotime, China). Vγ4T cell culture medium was used to resuspend cells to a density of 2.5 × 10^6^ cells/ml, cells were seeded on the preplate with Vγ4 antibody (BioXcell, USA) for 2 days. Then Vγ4T cells were changed the plate and cultured in Vγ4T cells medium without CD28 antibody and Vγ4 antibody. The cells were activated by treatment with anti-CD3 (1 µg/ml, BioXcell, USA) for 12h could be used for identification and use on the 7^th^ day after purification. Flow cytometry with antibodies targeting CD3, γδ and Vγ4 (Invitrogen, BioLegend, USA) were used to determine the purity of the cell population.

### Osteogenic Differentiation of allo-MSCs or HVEM-Expressing allo-MSCs Co-Cultured With Vγ4T Cells *In Vitro*

Vγ4T cells were stimulated with 1 μg/ml CD3 and co-cultured with allogeneic MSCs or HVEM-MSCs at a ratio of 4:1 for 72 hours. Morphological changes in MSCs were observed during co-culture. C57BL/6 mouse MSC-specific osteogenic differentiation complete medium (Cyagen Biosciences China) was used instead of culture medium. The medium was changed every 3 days, and cells were cultured for 12 days. Reverse transcription polymerase chain reaction (RT-PCR) and Western blotting were conducted as follows: Runx2 and osterix (OSX) levels were measured after osteogenic differentiation culture for 3 days; Col I levels were measured on day 6, and osteocalcin (OCN) levels were measured on day 9. MSCs were co-cultured with Vγ4T cells at ratios of 1:0, 1:1, 1:2, and 1:4, and osteogenic differentiation was observed on the 9th day of co-culture. Alkaline phosphatase (ALP) staining was evaluated using the BCIP/NBT alkaline phosphatase assay kit (Beyotime, China), and on the 12^th^ day after co-culture, Alizarin Red S staining (Cyagen, China) was used for evaluation. ImageJ software (National Institutes of Health, USA) was used to analyse the images of stained cells.

### RT-PCR

mRNA was extracted from WT MSCs and MSCs expressing HVEM using TRIzol reagent (Invitrogen, USA) at designated time points after osteoinduction. According to the manufacturer’s instructions, the total mRNA was isolated and purified using the RNeasy Mini Kit (Qiagen, USA) and then quantified using a Beckman Coulter DU-600 (USA). cDNA was synthesized from 2 μg of total RNA using a ThermoScript RT-PCR system (Invitrogen, USA). PCR was performed using ReverTra -Plus-™ (TOYOBO) and Mir-X miRNA qRT-PCR TB Green^®^ Kit (Takara) and run on an ABI7500 qPCR system (Applied Biosystems, USA). Gene-specific primer pairs: COLI: 5′—3′(forward) CAGAGGCGAAGGCAACA 5′—3′(reverse) GTCCAAGGGAGCCACATC; Runx2: 5′—3′(forward) GCACA AACATGGCCAGATTCA, 5′—3′(reverse) AAGCCATGGTGCCCGTTAG; Osterix: 5′—3′(forward) CCCCTC GCTCTCTCCTATT 5′—3′(reverse) TAGGCACTGGCAAAGGC; OCN: 5′—3′(forward) AGCAGGAGGGCA ATAAGGT 5′—3′(reverse) GCTTTAGGGCAGCACAGG; GAPDH: 5′—3′(forward) TGTTTCCTCGTCCC GTAGA 5′—3′(reverse) ATCTCCACTTTGCCACTGC. Glyceraldehyde-3-phosphate dehydrogenase (GAPDH) served as a housekeeping gene. The gene expression level was quantified from the standard curve and normalized to GAPDH gene expression.

### Western Blotting Analysis

Total protein was extracted by RIPA lysis buffer (1 ml cold lysis buffer with 10 μl phosphatase inhibitor, 1 μl protease inhibitor and 5 μl 100 mm PMSF). The protein content was determined by the BCA method. Equal amounts of protein (20 μg) per sample were separated by sodium dodecyl sulfate polyacrylamide gel electrophoresis (SDS-PAGE) through 10% gels and transferred to a 0.22 μm polyvinylidene fluoride (PVDF) membrane. The membrane was blocked for 1-2 hours in 5% bovine serum albumin (BSA) in Tris-buffered saline containing 0.1% Tween-20 (TBST). Then, antibodies targeting HVEM (1:1000, Santa Cruz Biotechnology, USA), COL I (1:500, Bioss, China), Runx2 (1:1000, Cell Signaling Technology, USA), OCN (1:1000, Bioworld, USA), OSX (1:1000, Abcam, USA) and GAPDH (1:2000, BioLegend, USA) were incubated with the membranes overnight at 4°C. After a series of washes, the membranes were incubated with secondary antibodies (anti-rabbit IgG or anti-mouse IgG, 1:5000) for 1 hour at room temperature. The signals on the membranes were visualized with an enhanced chemiluminescence kit (UK) and Quantity One software, and quantitative analysis of gray value of WB band was performed by ImageJ software.

### ELISA

The supernatant of cells co-cultured for 3 days was processed to detect the expression of IL-17a and IFN-γ with respective ELISA kits (Dakewe, China).

### Construction of Mutilation Fracture Models With TEBs

**Figure 1:**
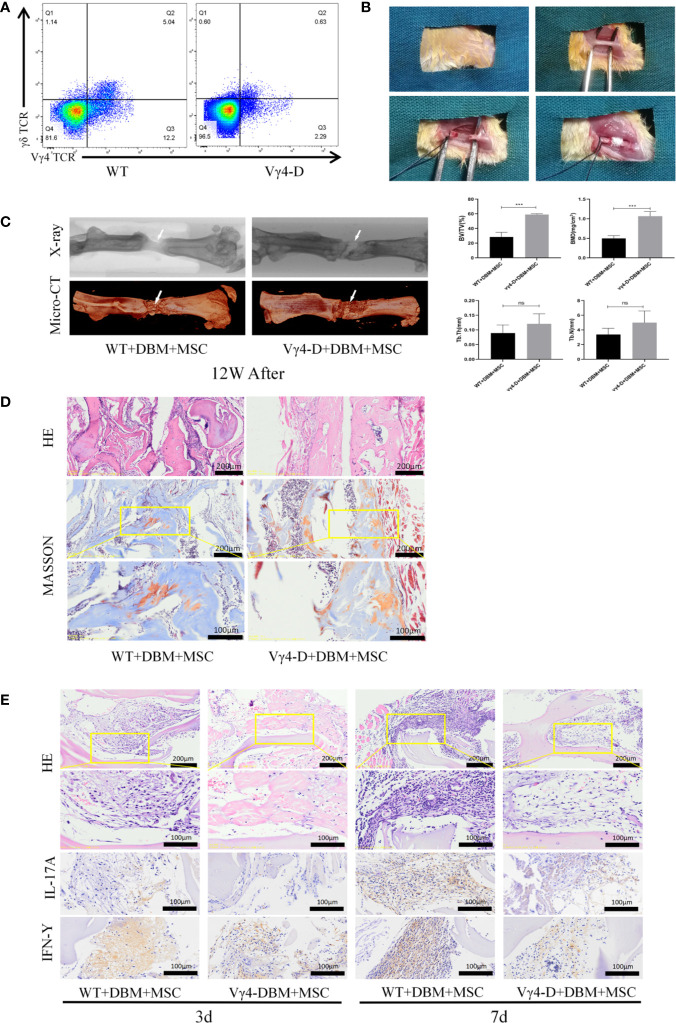
Establishment of femoral nonunion fracture model and Osteogenesis of TEB in vivo of femal Balb/c mice aged 8 weeks old. **(A)** Constructed Vγ4T cells free Balb/c mice (Vγ4-D). **(B)** Process for careting the nonunion fracture models in mice. **(C)** Morphological analysis of bone formation in 2 groups mice femur defect at 12 weeks after operation and Microarchitecture analyses of bone volume fraction (BV/TV) ,bone mineral density (BMD) , trabecular number of bone formation area (Tb.N) and  trabecular thickness (Tb.Th) by X-ray and 3D Reconstruction . Error bars represent mean ± SD. One-way ANOVA test was used to calculated P value (***P < 0.01, ns P > 0.05). **(D)** Histological analysis of newly formed bone by H&E and Masson’s Trichrome staining. **(E)** Infiltration of inflammatory cells and expressions of IL-17A and IFN-γ detected by immunohistochemistry at 3 days and 7 days post implantation.

The BMMSCs obtained above were inoculated onto both sides of demineralized bone matrix (DBM) that presented complete decalcification. Eight-week-old WT BALB/c female mice weighing 23-25 g were selected, 21 of which were treated with neutralizing antibody against Vγ4 (BioXcell, USA) at a dose of 200 μg/mouse (22). Three days later, usable mice lacking Vγ4T cells (Vγ4-D) were obtained (**Figure 1A**). Took out the spleen, ground it into a pulp, and lysed the remaining red blood cells, and the clearance of Vγ4T cells was detected by CD3, γδ and Vγ4 (Invitrogen, BioLegend, USA) levels (**Figure 1A**).

The mice were divided into groups according to mouse model and treatment as follows: WT/MSCs+DBM, Vγ4-D/MSCs+DBM, WT/MSCs-EGFP+DBM, WT/MSCs-HVEM+DBM and Vγ4-D/MSCs-HVEM+DBM, with 10 in each group. After mice were subjected to anaesthesia with 1% pentobarbital sodium, the right femur was exposed. A femoral shaft 2 mm long was cut off with a high-speed motor. The bone defect area was filled with constructed TEB, and the fracture area was fixed with an internal fixation pin to establish the mutilation fracture model (**Figure 1B**). Grafts were collected from each group at three days and seven days after surgery to histologically examine the inflammation and infiltration of the graft. The growth of new bone within the defect was evaluated by X-ray examination, micro-computed tomography (micro-CT) and histological analysis at 12 weeks post-operation.

### Micro-CT Measurement and Histological Assessment

Twelve weeks after the procedure, the femurs were removed for micro-CT scanning (quantum FX micro-CT imaging system, PerkinElmer, Ma, USA), and the three-dimensional images were reconstructed in the regions of interest (ROIs), which were defined as cylinders in the new bone in the non-union fracture area. New bone formation (bone volume/tissue volume ratio, BV/TV), bone mineral density (BMD), trabecular number (Tb.N) and trabecular thickness (Tb.Th), which were calculated with CTAn software (Bruker, Belgium), were recorded to evaluate bone regeneration and microstructure in bone defects.

The femurs of mice were fixed with 4% neutral-buffered paraformaldehyde, decalcified at 4°C for 4 weeks in 10% EDTA, embedded in paraffin, and sectioned at 5 μm. H&E and Masson’s trichrome staining was used to evaluate the tissue morphology.

### Immunohistochemistry

Immunohistochemistry was performed using SABC IHC kits (Zhongshan Corporation) with primary antibodies targeting IL-17a and IFN-γ (1:500, Abcam, USA) according to the manufacturer’s instructions, and nuclei were counterstained with haematoxylin. We captured the images with a Leica Microsystems microscope (DFC300 FX, Heerbrugg, Switzerland). The brief procedure for integrated optical density (IOD) analysis was performed as follows: an ROI for a positively stained area was analysed, and the average signalling intensity was quantified by ImageJ software.

### CCK8 Analysis

MSCs (1×10^4^ cells/well) or MSCs-HVEM (1×10^4^ cells/well) were cultured with or not with Vγ4T cells (4×10^4^ cells/well) in a Flat bottom 96-well plate for 3 days. The proliferation of cell was measured by using Cell Counting Kit-8 (CCK-8, Beyotime, China). Gently transferred the co-cultured medium into a new well,washed the 96-well plate with MSCs attachment 3 times with PBS added 100 μl new medium without FBS and also added 10 μl CCK8 solution to all wells, followed by incubation for 2 hours in the cell incubator. Finally, the absorbance value of the solution in each well was detected at the wavelength of 450 nanometers by a microplate reader (Type3001, Thermo Fisher Scientific, USA).

### Treatment of MSCs Co-Culture With Vγ4T Cells With IFN-γ, IFN-γ Antibody, IL-17A and IL-17A Antibody

In order to explore the possible mechanism that HVEM inhibits the secretion of IL-17A and IFN-γ in Vγ4T cells and promotes the osteogenesis of allo-MSCs, MSCs were planted in 6-well plates (1×10^5^ cells/well) and co-cultured with or without Vγ4T cells (4×10^5^ cells/well). Some wells added IFN-γ, IFN-γ antibody, IL-17A and IL-17A antibody (50 ng/ml, R&D Systems, USA), they were co-cultured or treated for 3d and incubated with osteogenic differentiation medium for another 9d, the expression of osteogenic markers was checked by Western blot analysis.

### Statistical Analysis

Data are presented as the means ± standard deviation (SD). Statistical analysis was performed with one-way analysis of variance (ANOVA) using the GraphPad Prism 8.0 statistical software package (GraphPad software, USA). The data conformed to the normal distribution when checked by Normality and Lognormality Tests, so we chose the ordinary ANOVA test or Brown-Forsythe and Welch ANOVA tests according to whether the data had the same SDs. P < 0.05 was considered statistically significant.

## Results

### Osteogenic Effect of TEBs Constructed by MSCs *In Vivo* in Vγ4T Cell-Free Mice

The methods of constructing TEB with MSCs were based on our previous studies (16), and TEB was transplanted into sites of femoral-amputated defects created by a high-speed motor. After morphological analysis of the mouse femur and TEB by micro-CT, the scan image and X-ray image at 12 weeks after surgery were reconstructed in three dimensions using CTVox software (**Figure 1B**). Nascent bone in the WT and Vγ4-D groups was observed in the fracture area. Upon assessment, we found that the quality of new bone in the Vγ4-D group was better than that in the WT group according to the BMD, BV/TV, Tb.N, and Tb.Th values (**Figure 1C**).

Twelve weeks after transplantation, H&E and Masson trichrome staining were used to further observe the degree of new bone formation in the transplanted area. More nascent recombinant woven calli formed in the Vγ4-D group than in the WT group. These results suggested that the osteogenic efficiencies of transplanted TEB were strengthened when Vγ4T cells were eliminated (**Figure 1D**). Additionally, at 3 days and 7 days after implantation, we observed the infiltration of inflammatory cells around the grafts with H&E staining and detected the expression of IL-17 and IFN-γ by immunohistochemical methods. The Vγ4-D group had less inflammatory cell infiltration and expressed fewer inflammatory mediators than did the WT group; therefore, we suggest that Vγ4T cells inhibit the osteogenic differentiation of grafts by secreting IL-17 and IFN-γ and subsequently inducing inflammation (**Figure 1E**).

### Osteogenic Differentiation of MSCs After Co-Culture With Vγ4T Cells *In Vitro*

**Figure 2:**
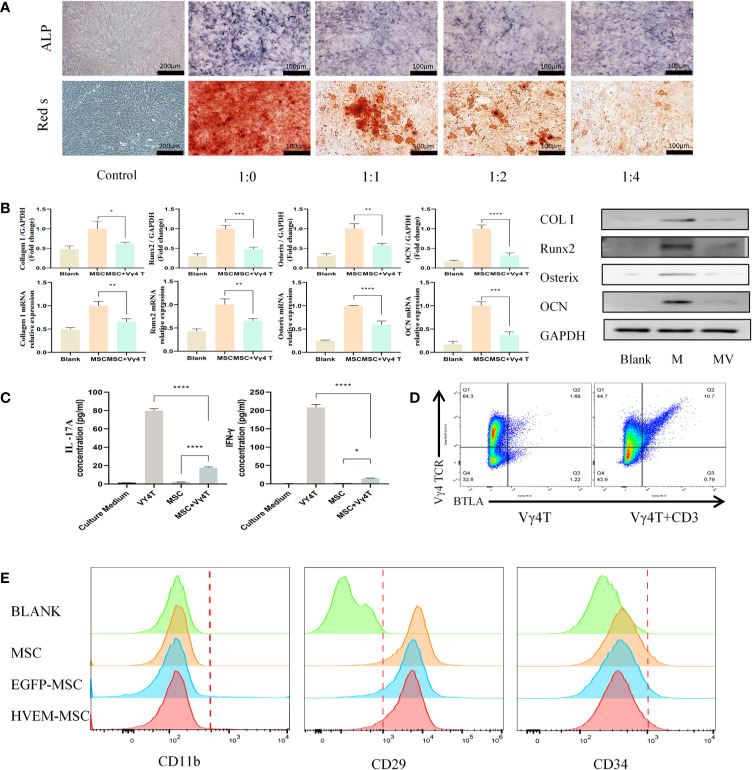
Vγ4T cells inhibite the osteogenic differentiation of MSCs. **(A)** ALP staining and alizarin red staining show various concentrations of Vγ4T cells inhibited the osteogenic differentiation of MSCs. **(B)** Expression of markers of osteogenesis by MSCs with or not with Vγ4T cells(the ratio is 1:4) were measured by RT-PCR and Western blotting analyses, relative protein expression of COL I, Runx2, Osterix, and OCN by Western blotting, mRNA expression levels were tested by RT-PCR. **(C)** The concentration of IL-17A and IFN-γ was measured in the supernatant of co cultured cells for 3 days. **(D)** BTLA expression in Vγ4T cells. **(E)** Changes of surface markers of MSCs or HVEM- expressing MSCs (MSCs-HVEM). Error bars represent mean ± SD. One-way ANOVA test was used to calculated P value (*P < 0.05, **P < 0.01, ***P < 0.001, ****P < 0.0001).

We co-cultured MSCs and Vγ4T cells at ratios of 1:0, 1:1, 1:2, and 1:4 for 3 days before we removed the supernatant, washed the cells 3 times with PBS and added osteoinduction medium, which was changed every 3 days. Cells were stained for ALP 9 days after osteoinduction and with Alizarin Red 12 days after osteoinduction. The experimental results showed that Vγ4T cells can inhibit the osteogenic differentiation of MSCs, and as the proportion of Vγ4T cells increased, the inhibitory effect was also strengthened (**Figure 2A**). To evaluate the osteogenic differentiation potential of MSCs after co-culture with Vγ4T cells (ratio of 1:4), RT-PCR and Western blot were used to measure the mRNA and protein expression, respectively, of osteogenic genes such as COL I, Runx2, OSTERIX, and OCN. We found that the mRNA and protein expression levels of osteogenic genes in MSCs co-cultured with Vγ4T cells in osteogenic induction medium were lower than those in MSCs cultured with osteogenic induction medium alone but higher than those in MSCs cultured with complete medium (**Figure 2B**). However, we do not know why Vγ4T cells could inhibit the osteogenic differentiation of MSCs *in vitro*. After detecting IL-17 and IFN-γ in the co-culture supernatant, we discovered that IL-17 and IFN-γ in the co-culture supernatant was suspected to downregulate the osteogenic differentiation of MSCs (**Figure 2C**). We also wanted to know whether inhibiting the function of Vγ4T cells would promote MSC bone formation.

### The Expression of BTLA (CD272) in Vγ4T Cells and Changes in Surface Marker Expression in HVEM-Expressing MSCs

FACS analysis showed of BTLA (CD272) expression in 10.9% of activated Vγ4T cells but only in 1.69% of inactivated Vγ4T cells (**Figure 2D**). Twenty-four hours after transfection of MSCs with Ad-EGFP-HVEM recombinant adenovirus, we used flow cytometry to analyse the expression of surface markers on MSCs, EGFP-MSCs cells, and HVEM-MSCs, and there was no difference in the expression of CD11b, CD29 and CD34 among the three groups (**Figure 2E**).

### Osteogenic Differentiation of HVEM-MSCs After Co-Culture With Vγ4T Cells *In Vitro*

**Figure 3:**
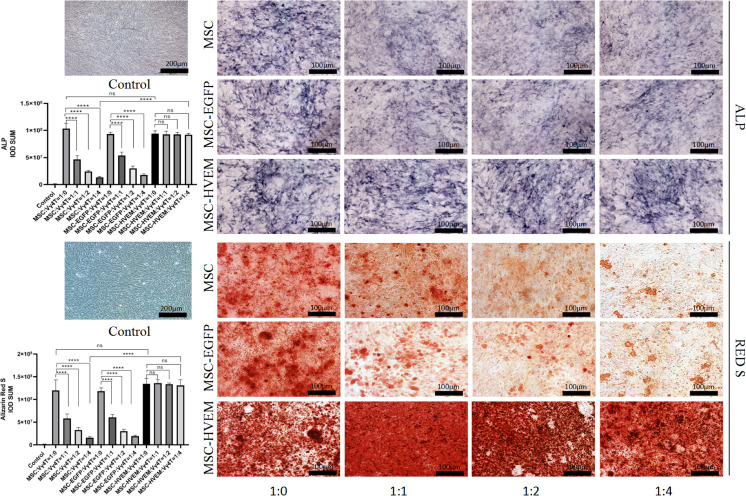
Vγ4T cells co-culture with MSCs or MSCs-HVEM then induced in osteogenic medium and ALP staining and alizarin red staining show various concentrations of Vγ4T cells inhibited the osteogenic differentiation of MSCs but MSCs-HVEM could reverse the inhibition of Vγ4T cells. Error bars represent mean ± SD. One-way ANOVA test was used to calculated P value (****P < 0.0001, ns P > 0.05).

**Figure 4:**
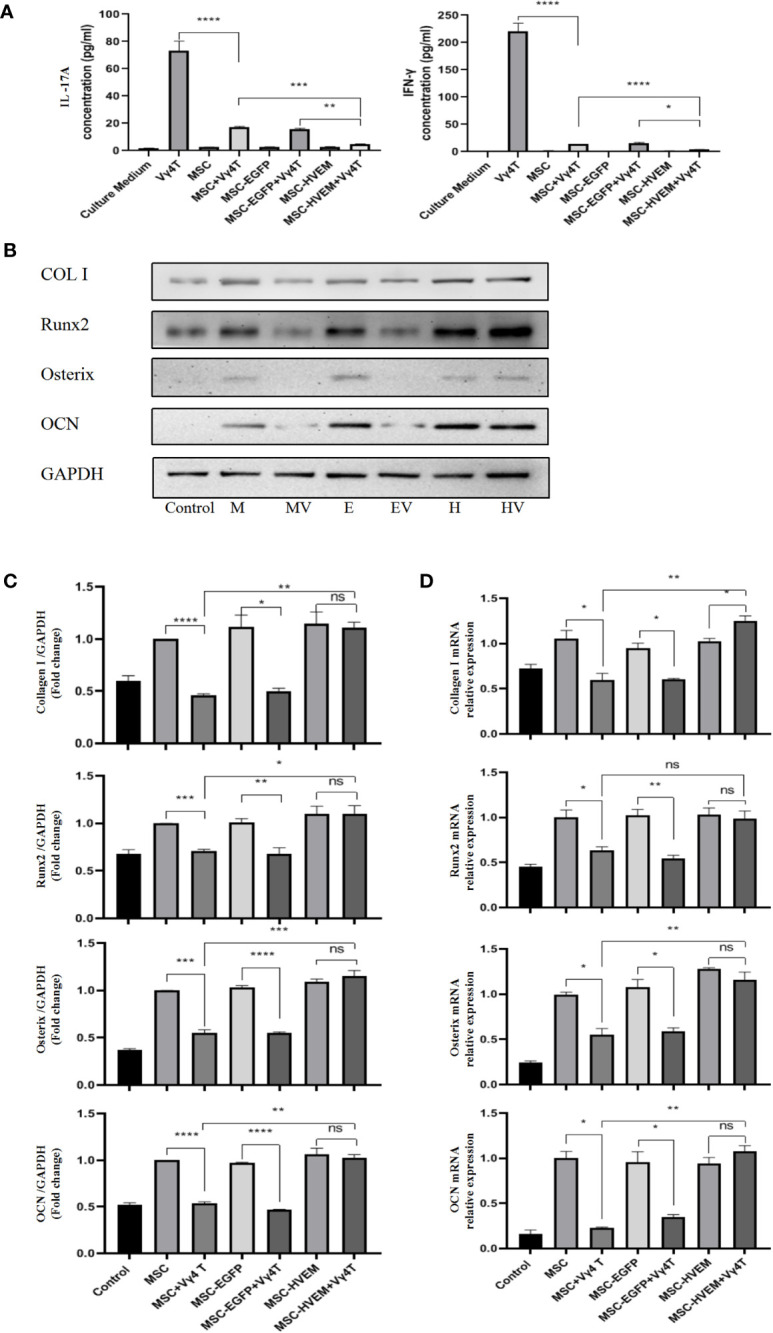
Vγ4T cells co-culture with MSCs or MSCs-HVEM then induced in osteogenic medium. **(A)** MSC can inhibit IL-17A and IFN-γ secretion from purified Vγ4T cells cells, the inhibition ability of MSCs-HVEM was stronger than that of MSC cells. **(B, C)** The Expression of markers of osteogenesis such as COL I, Runx2, Osterix, and OCN by MSCs or MSCs-HVEM  with or not with Vγ4T cells(the ratio is 1:4) were measured by Western blotting analyses, the osteogenic differentiation ability of MSC decreased when co-culture with Vγ4T cells but MSCs-HVEM can counter or even reverse that trend .MSCs (M),MSCs + Vγ4T cells (MV), MSCs-EGFP (E), MSCs-EGFP + Vγ4T cells (EV), MSCs-HVEM (H), MSCs-HVEM + Vγ4T cells (HV). **(D)** mRNA expression levelsof COL I, Runx2, Osterix, and OCN were tested by RT-PCR after 9 days of MSCs or MSCs-HVEM with or not with Vγ4T cells (the ratio is 1:4, relative to expression in MSCs showed the same trend. Error bars represent mean ± SD. One-way ANOVA test was used to calculated P value (*P < 0.05, **P < 0.01, ***P < 0.001, ****P < 0.0001, ns P > 0.05).

According to the ALP and Alizarin Red staining results, the osteogenic differentiation ability of MSCs transfected with adenovirus carrying the HVEM gene was roughly the same as that of MSCs transfected with empty virus and non-transfected MSCs. The presence of HVEM could resist the inhibitory effect of Vγ4T cells on MSC osteogenesis(**Figure 3**). The co-cultured supernatant was removed and tested for IL-17A and IFN-γ levels by ELISA; the results indicate that HVEM significantly inhibited the secretion of IL-17A and IFN-γ by Vγ4T cells (**Figure 4A**). This suggested that HVEM promotes MSCs osteogenic differentiation because HVEM inhibited the function of Vγ4T cells and reduced the secretion of IL-17a and IFN-γ. Subsequently, we used Western blot and RT-PCR to detect the expression of MSC-related osteogenic genes after osteoinduction and found that the osteogenic expression in MSCs and EGFP-MSCs co-cultured with Vγ4T cells was weakened, while the expression in HVEM-MSCs co-cultured with Vγ4T cells returned to normal levels (**Figures 4B–D**).

### Effects of MSCs-HVEM on Proliferation of MSCs and Vγ4T Cells

**Figure 5:**
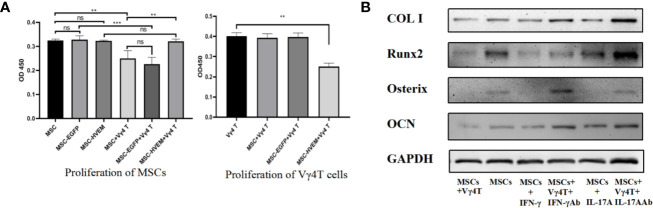
The effect of Vγ4T cells on MSCs proliferation and osteogenic differentiation. **(A)** The proliferation of cell was measured by using CCK-8 assay on 3^rd^ day. Absorbance values at 450 nm for each group are shown when MSCs or MSCs-HVEM co-culture with or not with Vγ4T cells. Data are reported as means ± SD. * 0.01 ≤ P ≤ 0.05, ** P ≤ 0.01, *** P ≤ 0.001 (One Way ANOVA). **(B)** The Expression of markers of osteogenesis such as COL I, Runx2, Osterix, and OCN by MSCs with Vγ4T cells (the ratio is 1:4) treated with IFN-γ, IFN-γ antibody (IFN-γ Ab) , IL-17A, IL-17A antibody (IL-17A Ab) are shown. Error bars represent mean ± SD. One-way ANOVA test was used to calculated P value (**P < 0.01, ***P < 0.001, ns P > 0.05).

To determine the effect of HVEM on proliferation of MSCs and Vγ4T cells, MSCs or MSCs-HVEM were co-cultured with or without Vγ4T cells (ratio was 1:4). The result of CCK8 test showed that MSCs proliferation was obviously inhibited when co-cultured with Vγ4T cells and HVEM-expressing MSCs could reverse this trend. Compared with MSCs and MSCs-EGFP, MSCs-HVEM could also inhibit the proliferation of Vγ4T cells (**Figure 5A**).

### IL-17 and IFN-γ Inhibited Osteogenesis of MSCs *In Vitro*

We co-cultured the MSCs and Vγ4T cells (ratio was 1:4) and with the treatment of IFN-γ, IFN-γ antibody (IFN-γ Ab), IL-17A and IL-17A antibody (IL-17A Ab) for 3 days, added osteoinduction medium, which was changed every 3 days. Western blot was used to detect the expression of MSC-related osteogenic genes, we found that treatment inhibited osteogenic differentiation of MSCs *in vitro*, it was the same as treatment with Vγ4T cells, added the antibody of IL-17 or IFN-γ into the co-culture system, the osteogenic differentiation of MSCs was better than those co-cultured with Vγ4T cells (**Figure 5B**).

### Osteogenesis Repair Function of MSCs-HVEM in Bone Defect Areas *In Vivo*

**Figure 6:**
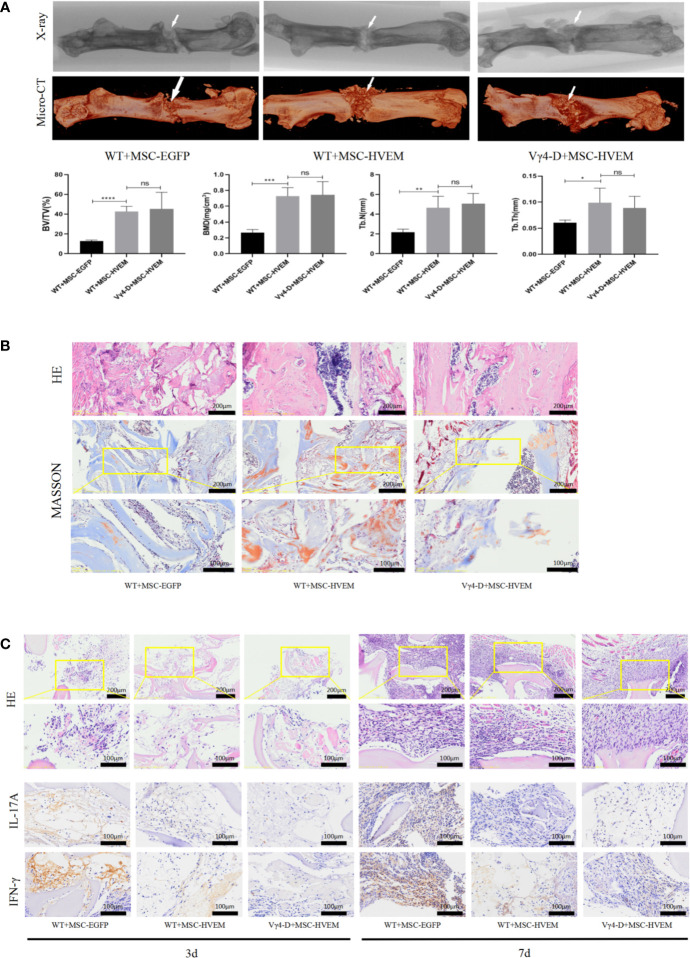
Osteogenic ability and Local immune response of TEB constructed by MSC^HVEM^ in vivo. **(A)** Both WT+MSC^HVEM^ groups and VY4-D+MSC^HVEM^ groups show higher quality of bone formation than that of WT+MSC in BV/TV, BMD, Tb.N, Tb.Th, but the quality of bone formation of between WT+MSC^HVEM^ groups and VY4-D+ MSC^HVEM^ groups had no statistics difference (P > 0.05). **(B)** Histological analysis of newly formed bone by H&E and Masson’s Trichrome staining in 3 groups. **(C)** Infiltration of inflammatory cells and expressions of IL-17A and IFN-γ detected by immunohistochemistry at 3 days and 7 days post implantation. Error bars represent mean ± SD. One-way ANOVA test was used to calculated P value (*P < 0.05, **P < 0.01, ***P < 0.001, ****P < 0.0001, ns P > 0.05).

Based on morphological analysis of mouse femur and TEB by micro-CT, 3D reconstruction of scan image and X-ray image using CTVox software, new bones could be seen in each group in the fracture area 12 weeks after surgery (except for the samples with bending fractures). Comparing the HVEM-expressing MSCs WT mouse group (WT+MSCs-HVEM) and HVEM-expressing MSCs Vγ4T cell-free mouse group (Vγ4-D+MSCs-HVEM), there were similar osteogenesis performances in terms of new bone volume, new bone density, number of bone trabeculae, and average trabecular bone thickness; any differences were not statistically significant. Both groups showed better osteogenesis performance than did the EGFP-MSCs WT mouse group with respect to the same variables (**Figure 6A**). This meant that in the presence of Vγ4T cells, TEB osteogenesis was weakened, while HVEM expression on MSCs or clearance of Vγ4T cells reduced inflammation and restored TEB osteogenesis. The levels of bone repair in the TEB area were analysed by immunohistochemical analysis using H&E and Masson’s trichrome staining at 12 weeks after implantation, which showed the same tendency (**Figure 6B**).

We also harvested bone grafts 3 and 7 days after implantation and performed H&E staining after pathological sectioning. The numbers of inflammatory cells in the HVEM-MSCs WT mouse groups and HVEM-MSCs Vγ4T cell-free mouse groups were both lower than those in the EGFP-MSCs WT mouse group, which indicated that HVEM-transformed MSCs could inhibit early inflammation, possibly by controlling Vγ4T cell function. The HVEM-MSCs WT mouse group and HVEM-MSCs Vγ4T cell-free mouse group expressed lower levels of inflammatory mediators than did the EGFP-MSCs WT mouse group based on immunohistochemical detection of IL-17A and IFN-γ expression (**Figure 6C**).

## Discussion

TEB repair of bone defects requires efficient seed cells as a core. At present, many seed cell studies involve MSCs with osteogenic differentiation abilities (23, 24). Unfortunately, autologous MSCs (auto-MSCs) are short in number, have a long preparation time, and cause donor area-related disease. By contrast, allo-MSCs provide the ability of pluripotent differentiation and the ability to overcome the abovementioned difficulties. Many scholars have shown that allo-MSCs from sheep, rats and mice are immunogenic *in vivo*, which may lead to graft failure without immunosuppressive therapies (7–9, 25, 26). Therefore, due to potential immune rejection, the clinical use of allo-MSCs is still controversial. The balance of inflammation is essential to regulate the osteogenesis of TEB to repair bone defects. Our previous studies have found that HVEM-modified MSCs promote TEB bone formation *in vivo (* 16), but the relevant mechanism is still unclear. Vγ4T cells can promote acute rejection through IL-17 signalling and reduce the survival time of mice with transplanted hearts and skin (13). We speculate that HVEM inhibits the function of Vγ4T cells to promote MSCs bone formation *in vitro* and *in vivo*. In this study, we tried to determine the role of Vγ4T cells in TEB osteogenesis by eliminating Vγ4T cells and explored whether HVEM-modified MSCs can inhibit the function of Vγ4T cells and promote osteogenesis.

Our results showed that TEB osteogenesis was enhanced after Vγ4T cells were eliminated. In the early stage of implantation in Vγ4-D mice, the infiltration of inflammatory cells and the secretion of inflammatory factors in the implant area were reduced. Compared with allogeneic MSCs alone, allogeneic MSCs co-cultured with Vγ4T cells showed significantly reduced expression levels of the osteogenic markers COLI, Runx2, OSX, and OCN as well as lower numbers of mineralized nodules and reduced ALP activity when cultured in osteogenic medium. The concentrations of IL-17 and IFN-γ in the co-culture supernatant were higher than those in the MSCs monoculture supernatant. IL-17A and IFN-γ treatment inhibited osteogenesis of MSCs *in vitro.* These results confirmed our speculation that Vγ4T cells could inhibit MSCs osteogenic differentiation, possibly by secreting IL-17 and IFN-γ. These results are consistent with the findings of Spaggiari et al. (27–29). By contrast, compared with allogeneic MSCs under the same conditions, MSCs expressing HVEM maintained the expression of osteogenic markers, ALP activity and calcium nodule formation, thereby maintaining their osteogenic differentiation ability. HVEM-transfected MSCs inhibited the secretion of IL-17 and IFN-γ from Vγ4T cells and enhanced the osteogenic ability of TEB seed cells. These results are consistent with the findings of Dai et al., who confirmed that CTLA4- or HVEM-modified MSCs showed good heterotopic osteogenesis after subcutaneous implantation or transplantation into the femoral muscle pocket of rats as well as excellent repair effects on large orthotopic bone defects after xenotransplantation (1, 16, 30).

The mechanism by which HVEM suppresses the immune response and promotes osteogenesis has not been fully elucidated. Our previous study found that IL-17 treatment of MSCs can inhibit their osteogenic differentiation, but the presence of HVEM on MSCs can reverse this trend (16). Vγ4T cells, a cell type that plays an important role in immune diseases, is the main source of IL-17 in the early stage of the immune response (12, 31), and we observed that MSCs expressing HVEM significantly inhibited IL-17 and IFN-γ secretion from Vγ4T cells and the proliferation of Vγ4T cells when co-cultured, and also released the inhibitory effect of Vγ4T cells on MSCs proliferation during co-cultured. IL-17 not only is an effective stimulator of RANKL expression but also induces the synthesis of matrix-degrading enzymes to reduce the bone formation of MSCs (32, 33). Kim et al. found that rat calvarial cells express IL-17-related receptor subtypes (34). IFN-γ can enhance not only the immunogenicity of MSCs by increasing the expression of the major histocompatibility complex (MHC-II) (35) but also TNF-α signalling, leading to MSCs apoptosis and a decrease in osteogenic differentiation (27, 36). BTLA-HVEM, CTLA4 and PD1 are all negative immune cell regulators. Among them, only BTLA is highly expressed on γδT cells, thereby inhibiting the proliferation and function of T cells and thus playing an important role in the immune response (37). HVEM can induce inhibitory signal transduction of lymphocytes and innate immune cells through BTLA (38). Our study showed that Vγ4T cells with CD3 stimulation could significantly enhance the expression of BTLA (**Figure 2D**). Through interaction between HVEM on MSCs and BTLA on Vγ4T cells, HVEM-expressing MSCs could inhibit Vγ4T cells to secrete IL-17 and IFN-γ, resulting in decreased inflammation and increased bone formation. It’s reasonable to assume that the activation of BTLA *via* HVEM results in the consequent down-regulation of the NF-κB signalling pathway of Vγ4T cells (39, 40), thereby inhibiting IL-17A and IFN-γ secretion to promote effective bone formation. Additional studies by scholars have shown that the STAT3 and nuclear factor (NF)-κB signalling pathways and the key transcription factor RORγt strictly regulate the secretion of IL-17 (40). Chang et al. stated that the proinflammatory cytokine IL-17 stimulated the IκB kinase (IKK)-NF-κB signalling axis, thereby reducing the osteogenic differentiation of MSCs. By contrast, inhibiting IKK-NF-κB (IKKVI) signal transduction could significantly enhance MSC-mediated bone formation (41). The infiltration of inflammatory cells mediated by the proinflammatory factor IFN-γ was also negatively correlated with the late ossification of the grafts (42). Therefore, in allogeneic transplantation, after Vγ4T cells were cleared, the level of early inflammation, which promoted the effective formation of bone, was reduced. Of course, our study just demonstrated that Vγ4T cells were deeply involved in HVEM-mediated pro-osteogenesis of MSCs. The precise roles of Vγ4T cells in HVEM-mediated pro-osteogenesis of MSCs need to be further clarified by using animals with conditional depletion of BTLA in Vγ4T cells. Furthermore, Vγ4T cells as early major source of IL-17A and IFN-γ played an important role in initiating inflammation. However, the exactly effects of IL-17A and IFN-γ on MSCs osteogenesis had not been confirmed *in vivo*. IL-17A ^−/−^ or IFN-γ ^−/−^ mice should be used to answer this question in future study.

In conclusion, the current research shows that clearing Vγ4T cells in mice can reduce inflammation in the graft area and significantly improve graft osteogenesis. In addition, allo-MSCs expressing HVEM can also inhibit the function of Vγ4T cells *in vitro*, inhibit the proliferation of Vγ4T cells *in vitro*, release the inhibitory effect of Vγ4T cells on MSCs proliferation during co-culture, thereby reducing localized inflammation and enhancing graft osteogenesis. Our results provide a solid foundation for exploring HVEM-expressing MSCs as an ideal source of seed cells for xenogeneic bone transplantation in tissue engineering methods.

## Data Availability Statement

The raw data supporting the conclusions of this article will be made available by the authors, without undue reservation.

## Ethics Statement

The animal study was reviewed and approved by the Animal Ethics Committee of the Third Military Medical University.

## Author Contributions

Conceptualization: FD and WH. Methodology: LH, JX, LS, ZR, and RZ. Validation: LH, JX, LS, ZR, and RZ. Formal analysis: LH, JX, and LS. Investigation: LH and ZR. Resources: LH, JX, LS, ZR, and RZ. Writing-original draft: LH and JX. Writing-reviews and editing: FD and WH. Funding acquisition: FD. Supervision: FD and WH. All authors contributed to the article and approved the submitted version.

## Funding

The study was supported by grants from the National Natural Science Foundation of China (no. 81874005).

## Conflict of Interest

The authors declare that the research was conducted in the absence of any commercial or financial relationships that could be construed as a potential conflict of interest.
