# Supplementary figures and images for: HVEM Promotes the Osteogenesis of allo-MSCs by Inhibiting the Secretion of IL-17 and IFN-γ in Vγ4T Cells
Source: Front Immunol. 2021 Jun 23;12:689269. doi: 10.3389/fimmu.2021.689269 (PMC8261146; doi:10.3389/fimmu.2021.689269)

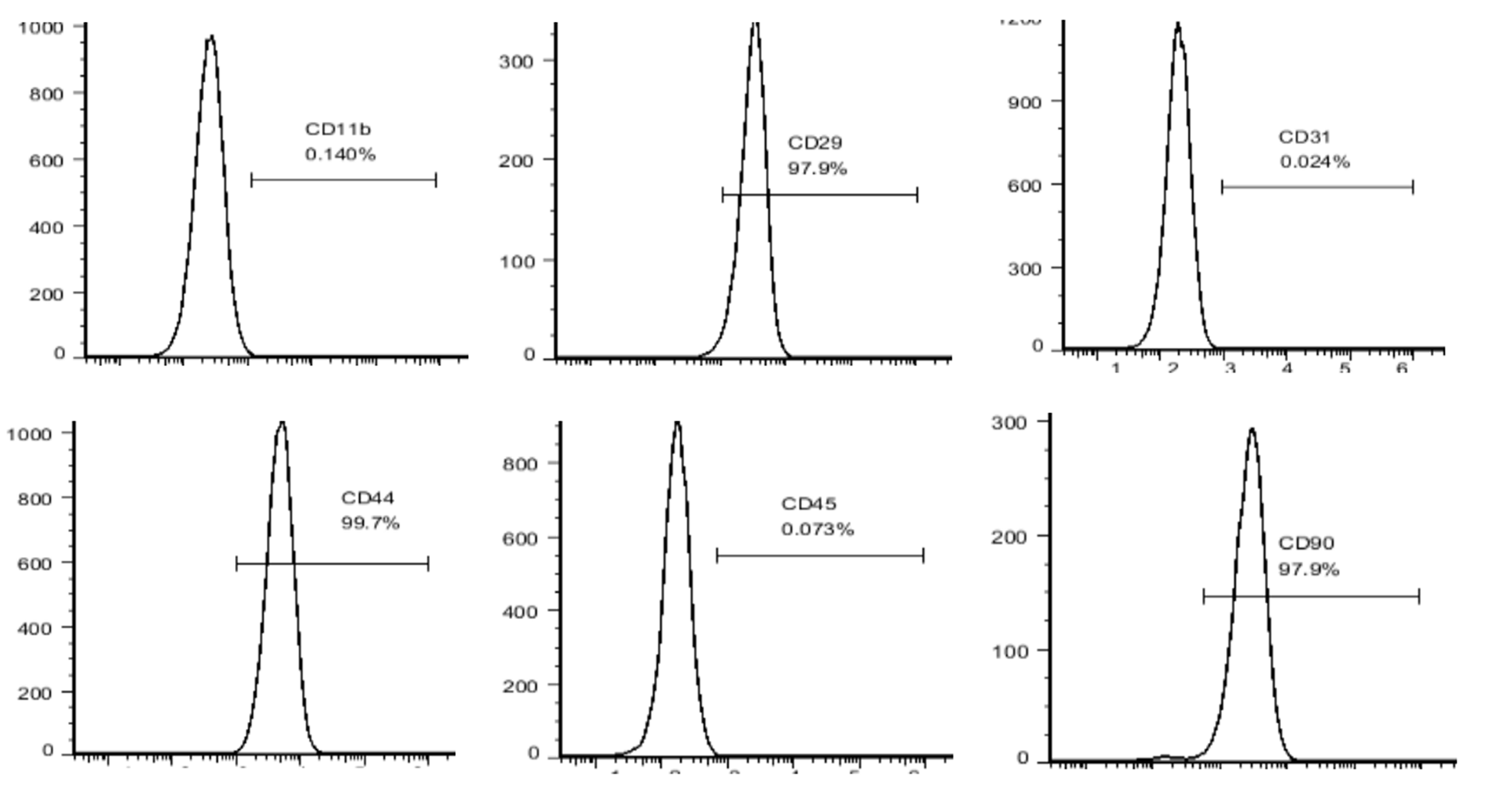

Supplement: Supplementary file 1 [file Image_1.tif]

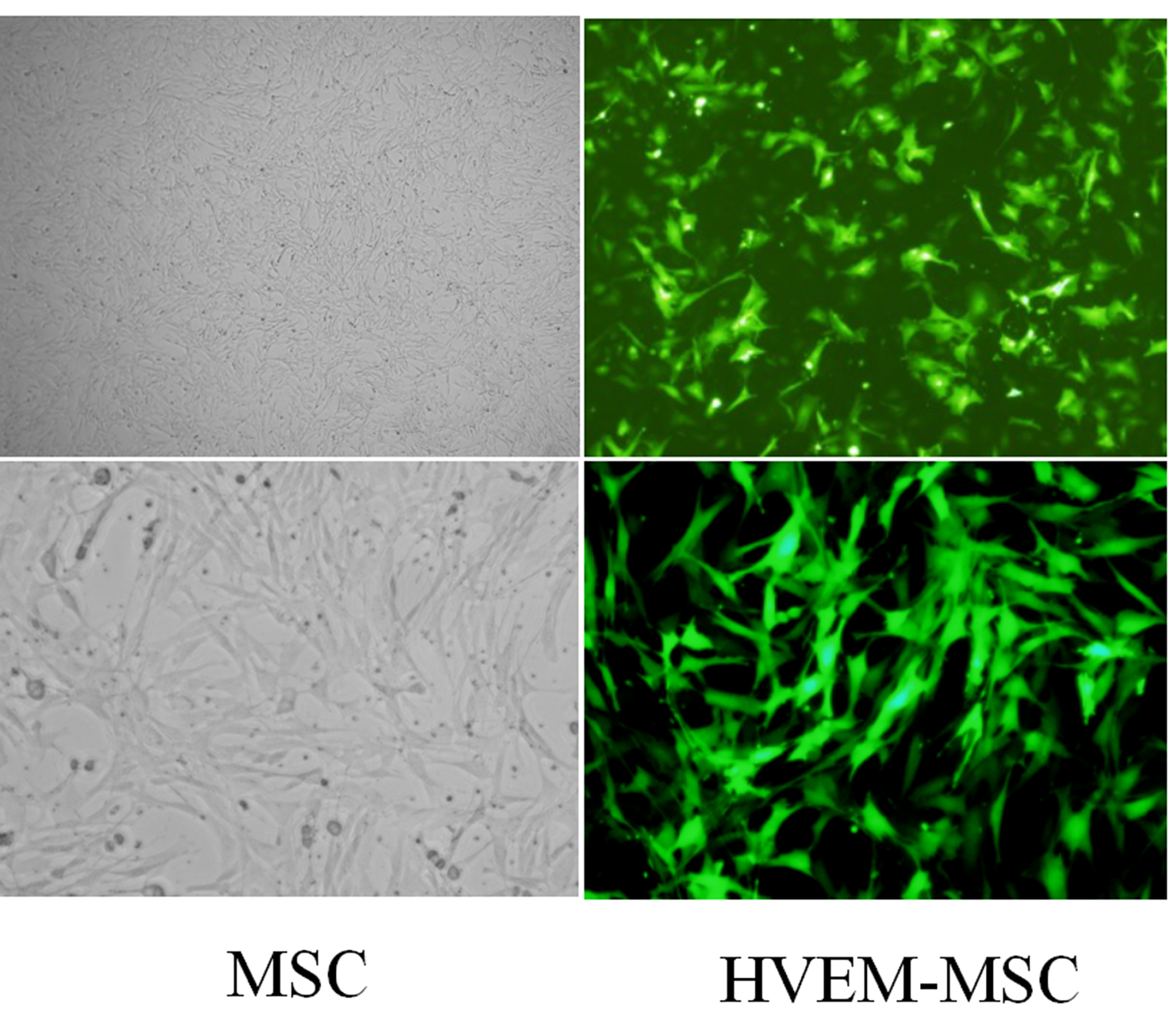

Supplement: Supplementary file 2 [file Image_2.tif]

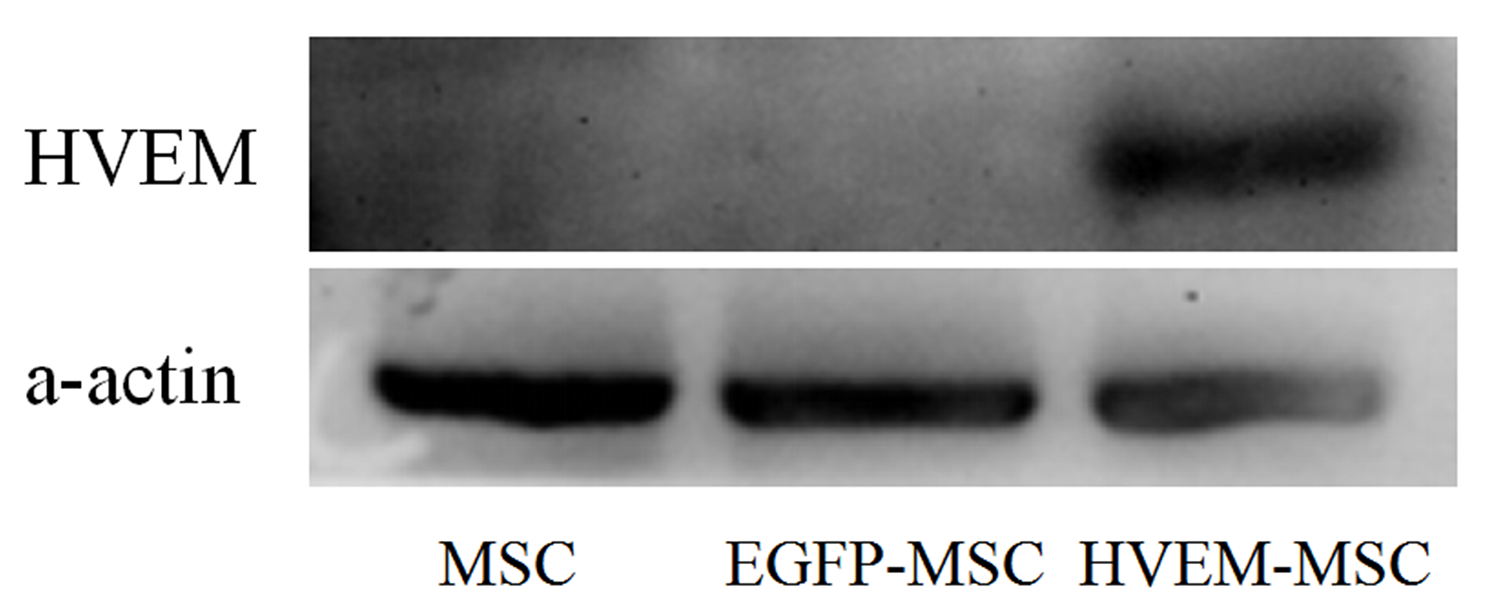

Supplement: Supplementary file 3 [file Image_3.tif]

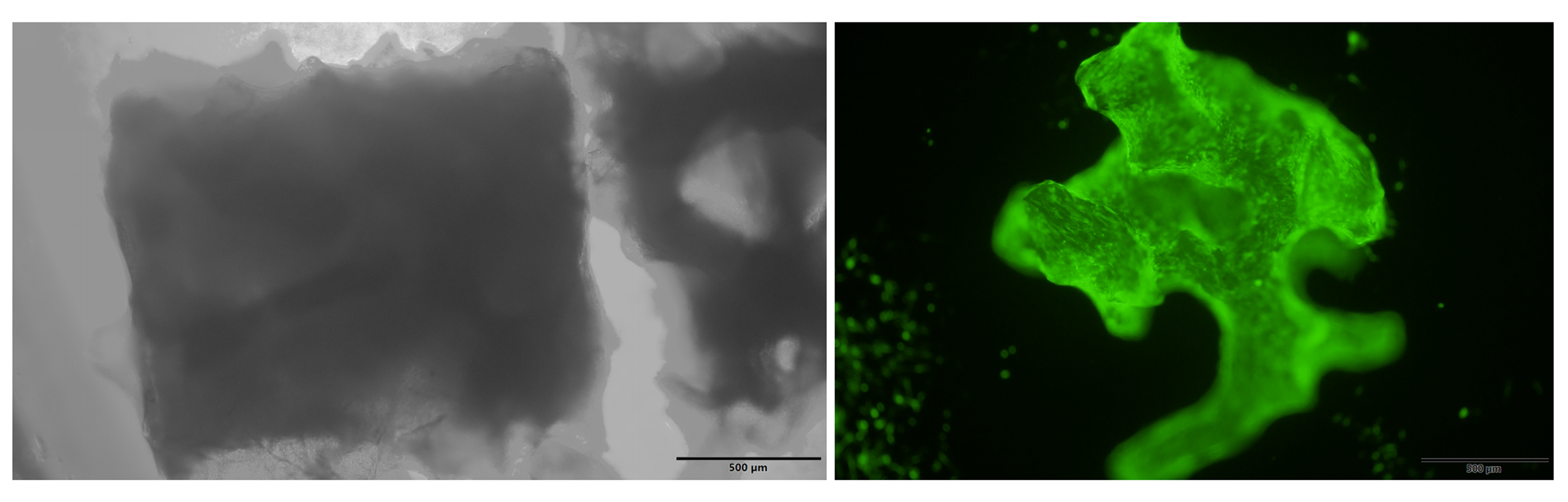

Supplement: Supplementary file 4 [file Image_4.tif]

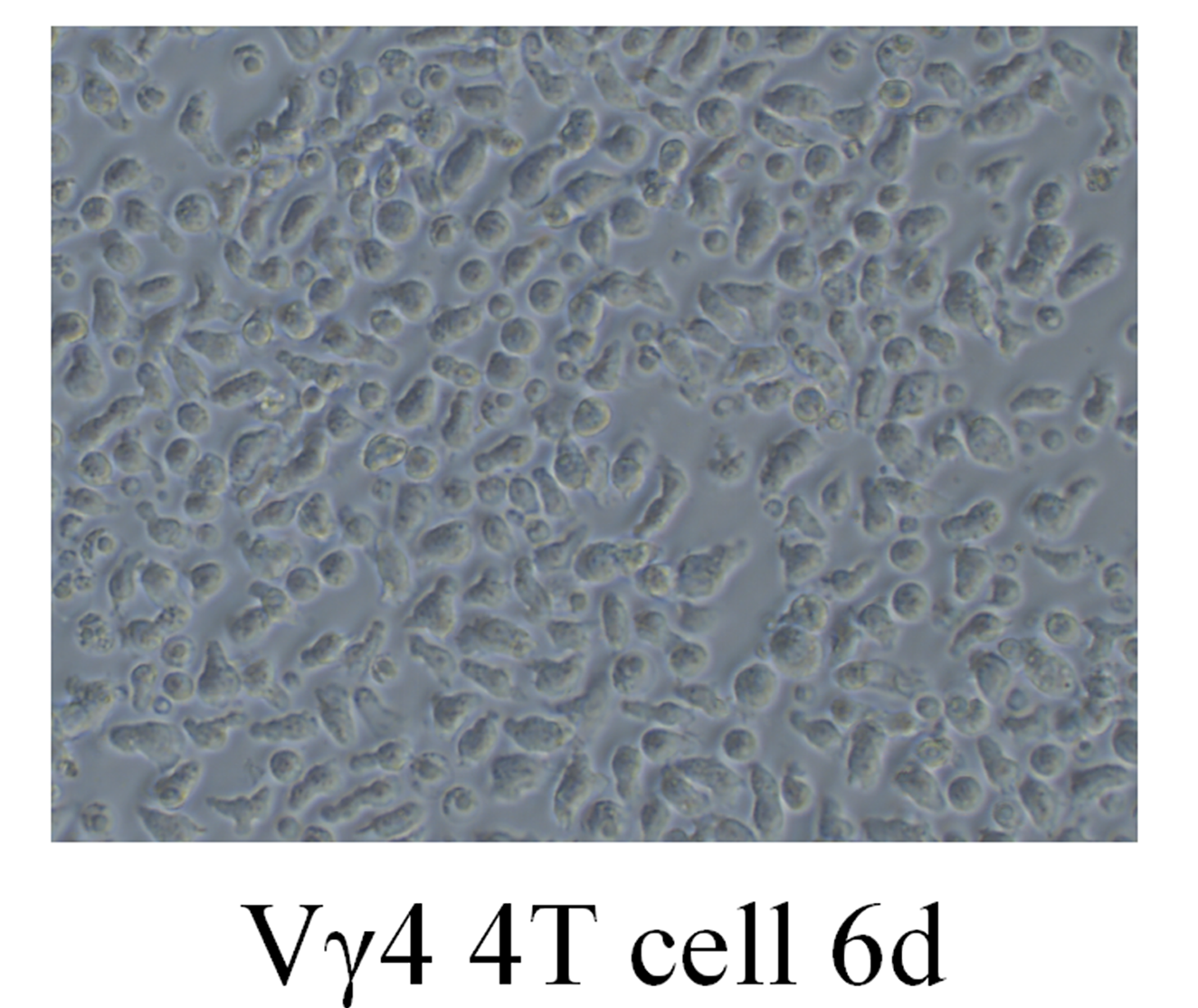

Supplement: Supplementary file 5 [file Image_5.tif]
